# Figure-of-eight bandage versus arm sling for treating middle-third clavicle fractures in adults: study protocol for a randomised controlled trial

**DOI:** 10.1186/s13063-016-1355-8

**Published:** 2016-05-04

**Authors:** Mario Lenza, Luiz Fabiano Presente Taniguchi, Mario Ferretti

**Affiliations:** Hospital Israelita Albert Einstein, Avenida Albert Einstein, 627/701 – Jardim Leonor, CEP: 05652-900 São Paulo, SP Brazil; Hospital Municipal Dr. Moyses Deutsch, Estrada do M’boi Mirim, 5203 – Jardim Angela, CEP: 04948-030 São Paulo, SP Brazil

**Keywords:** Clavicle, Fracture healing, Fractures, Bone, Treatment outcome, Pseudarthrosis, Conservative treatment

## Abstract

**Background:**

Fracture of the clavicle is common, accounting for 2.6 to 4.0 % of all fractures, with an overall incidence of 36.5 to 64 per 100,000 per year. Around 80 % of clavicle fractures occur in the middle third of the clavicle. Randomised controlled trials comparing treatment interventions have failed to indicate the best therapeutic practices for these fractures. The objective of this study is to evaluate the effects (benefits and harms) of two commonly-used conservative interventions: the figure-of-eight bandage versus the arm sling as treatments of middle-third clavicle fractures.

**Methods/design:**

This project has been designed as a single-centre, two-arm randomised controlled trial that will compare two interventions: figure-of-eight bandage versus the arm sling. We propose to recruit 110 adults, aged 18 years or older, with an acute (less than 10 days since injury) middle-third clavicle fracture. The primary outcomes to be evaluated will be function and/or disability measured by the Disability of the Arm, Shoulder, and Hand (DASH) questionnaire. In order to assess the secondary outcomes, the Modified University of California at Los Angeles (modified – UCLA) Shoulder Rating Scale will be used. The occurrence of pain (Visual Analogue Scale for pain (VAS)), treatment failure, adverse events and the ability to return to previous activities will also be recorded and evaluated as secondary outcomes. Data analysis*:* the primary outcome DASH score and the secondary outcomes – modified UCLA and VAS scores – will be analysed graphically. We will apply generalised mixed models with the intervention groups (two levels), and time-point assessments (seven levels) as fixed effects and patients as a random effect.

**Discussion:**

According to the current literature there is very limited evidence from two small trials regarding the effectiveness of different methods of conservative interventions for treating clavicle fractures. This is the first randomised controlled trial comparing the figure-of-eight bandage versus the arm sling for treating clavicle fractures that follows the CONSORT Statement guidelines.

**Trial registration:**

ClinicalTrials.gov NCT02398006.

**Electronic supplementary material:**

The online version of this article (doi:10.1186/s13063-016-1355-8) contains supplementary material, which is available to authorized users.

## Background

Fracture of the clavicle accounts for 2.6 to 4 % of all fractures, with an overall incidence of 36.5 to 64 per 100,000 per year [1–3]. The incidence is bimodal with peak incidence in youth and in later life with a male excess in the young and a female excess (7:1) in later life [1]. The most common site of fracture is the middle third of the clavicle, representing approximately 80 % of all clavicle fractures [3, 4].

**Fig. 1:**
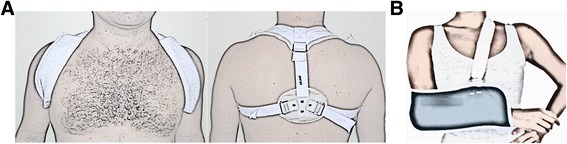
Conservative management to clavicle fractures: **a** Figure-of-eight bandage. **b** Simple arm sling

Traditionally, middle-third clavicle fractures have been treated conservatively, even when substantially displaced [5, 6]. A large number of methods to immobilise the region have been described. However, the most common methods are the use of a figure-of-eight strap (Fig. 1a), an arm sling (Fig. 1b), or a combination of the two [4, 7–9]. Indications for surgery include open fractures, severe displacement caused by comminution, suspicion of imminent skin lesion production by a sharp clavicle fracture edge, or neurovascular injuries. Relative indications for surgery include polytrauma, floating shoulder, painful malunion and painful nonunion. More recently though, the scope of indications has widened to include high-energy fractures, such as clavicle shortening of greater than 20 mm, complete displacement and severe comminution. There are several fixation techniques that can be used [10–13]. The most frequently used is internal fixation with plates or wires. Bone grafting may also be used [4, 9, 14, 15].

In contrast to fractures at other skeletal sites, there are only few randomised trials which have compared either conservative or surgical approaches to the management of clavicle fracture. In addition, the available evidence from isolated trials is limited regarding the effectiveness of the different methods of surgical and conservative interventions [16–18].

Lenza et al. [16] concluded that evidence of only two clinical trials published in the 1980s [7, 19] is insufficient to determine the effects (benefits and harms) of the different methods of conservative (non-operative) treatments for acute (treated soon after diagnosis) middle-third clavicle fractures in adults. A search strategy, updated in January 2014, did not find any new comparative studies on conservative interventions for treating clavicle fractures.

Current literature reinforces the view that the best conservative treatment for middle-third clavicle fractures in adults is controversial. A recent survey indicates that most US surgeons prefer to use a simple sling rather than the figure-of-eight bandage for conservatively treating their patients (94 % prefer the simple sling versus 6 % prefer the figure-of-eight bandage) [20]. In contrast, a survey on conservative treatment of clavicle fractures in Germany concluded that the simple clavicle fractures are treated in a nonsurgical way and orthopaedic surgeons prefer the use of the figure-of-eight bandage in 88 % of cases [21].

Therefore, there is a need to investigate which would be the best intervention (considering functional endpoints and adverse effects) in patients with clavicle fractures. Since nonsurgical treatments are the most prevalent in current clinical practice, the authors of this study chose to evaluate the effects (benefits and harms) of conservative interventions: the figure-of-eight bandage versus the arm sling, for treating middle-third clavicle fractures.

## Methods/design

**Fig. 2:**
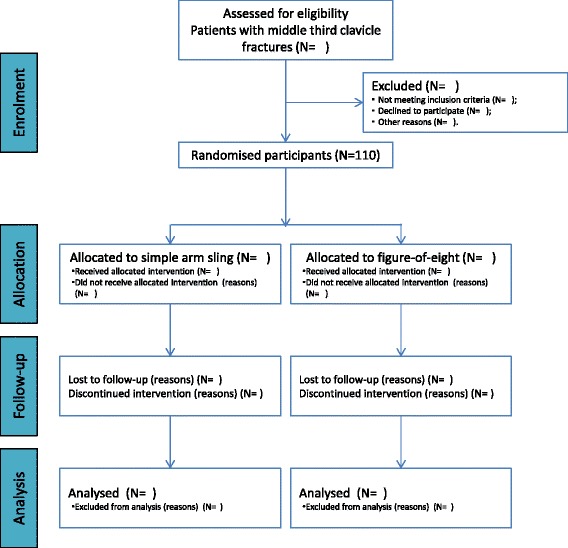
Flow of participants. Diagram shows the planned flow of participants through each stage of the study

This project has been designed as a single-centre, two-arm (parallel) randomised controlled trial with follow-up at 12 months, following the Consolidated Standards of Reporting Trials (CONSORT) Statement [22–25] (Fig. 2 shows the participant flow diagram). This study will be carried out at Hospital Municipal Dr. Moyses Deutsch in São Paulo, Brazil (a medium-complexity, public tertiary hospital), between May 2015 and March 2017. This study was approved by the ethical committee (CEP Hospital Israelita Albert Einstein/Plataforma Brasil 1966-14) and was registered in the ClinicalTrials.gov database (NCT02398006).

The trial will last a total of 26 months. The initial 2 months will be a pre-trial period to be followed by a recruitment period of 10 months. The subsequent 12 months will cover the treatment and follow-up periods (these will overlap the recruitment phase) and a final 2 month period will be dedicated to data analysis.

### Participants

To be eligible, participants must meet the following criteria:

Inclusion criteria:

- Adults aged between 18 and 65 years with a middle-third clavicle fracture
- Acute fracture (less than 10 days), comprising all types of middle-third clavicle fractures (non-displaced and displaced fractures)
- No medical contraindication to proposed methods of immobilisation
- Understanding of the Portuguese language and providing written informed consent

Exclusion criteria:

- Pathological fracture
- Open fracture
- Neurovascular injury on physical examination
- Associated head injury (Glasgow Coma Scale score <12)
- Ipsilateral upper limb fractures and/or dislocation (except of the hand and fingers)
- History of frozen shoulder
- Previous disease in the limb that could influence the results (e.g. rheumatoid arthritis)
- Inability to comply with follow-up (inability to read or complete forms)

### Interventions

Group 1: the orthopaedic surgeon will apply a figure-of-eight bandage (UDINE®) (Fig. 1a) – no attempt will be made to reduce the fracture. The figure-of-eight bandage will be used for 4 weeks, and every week the participants will return for checking and adjusting the immobilisations. In this way, the dominant arm can remain free and simple activities will be allowed (writing, keyboarding and other). Participants and relatives of participants will also be educated on how to tighten the bandage when necessary. The figure-of-eight bandage should be adjusted so that the shoulder is pressed back in an arched position, almost like a back-stretch.

The figure-of-eight should be used all day (including bath-time and bed-time); participants will be instructed not to remove the immobilisations. Patients will be instructed on how to properly wear and adjust the immobilisations should they slacken or be removed from the shoulders. The following fitting instructions will be given:

1. Place arms through arm straps so that the felt pad is located on the back and the foam insert is placed between the shoulder blades
2. Adjust by pulling the arm straps tight. Fasten the contact closures on the arm pad
3. The cushioned arm pad should be adjusted so that it fits under the arm and in front of the shoulder. The straps should be tight enough to pull the shoulders back

A sponge bath or a tub bath will be recommended. Patients will also be advised that during the night a rolled up towel can be placed on the bed between their shoulder blades. This helps to keep the shoulder in the correct position while asleep.

After 4 weeks, participants will be encouraged to discard the bandage. Load bearing will not be allowed before osseous consolidation (around 10 weeks).

Group 2: the orthopaedic surgeon will apply a standard arm sling (UDINE®) (Fig. 1b) that will be used for 4 weeks. The upper limb will be immobilised in internal rotation. The arm sling goes from the elbow of the injured arm, across the back to the patient’s opposite shoulder. The patient’s hand should be at or above the level of his/her elbow. Participants will be instructed to flex and extend their elbows for 10 minutes, three times a day. The patient will be allowed to remove the sling during bath-time. Load bearing will only be allowed after 10 weeks following the intervention.

All participants (of the two groups) will be placed on the standard analgesia protocol at our institution, which consists of patient-controlled ingested paracetamol (3 g/day) and/or sodium diclofenac (150 mg/day). On the final day of analgesic treatment (7 days), the total consumption of each drug will be recorded.

#### Care programme

Identical rehabilitation will be carried out in both intervention groups. After 4 weeks of treatment, a senior physiotherapist will instruct the participants on some simple standard home stretching exercises, which should be done daily for 30 minutes per day; these will be forward elevation and external rotation stretches and Codman pendulum exercises [26].

#### Assessment

After signing the consent form (Additional files 1 and 2), the researchers will collect the demographic data from all patients using a pre-prepared form (Additional file 3). Baseline data collected will include: age, gender, weight, height, addictions (e.g. smoking or alcohol), clinical comorbidities, occupation and mechanism of trauma. All fractures will be classified using the Edinburgh classification proposed by Robinson et al. [27].

### Outcomes

#### Primary outcomes

Function or disability will be measured by the Disability of the Arm, Shoulder, and Hand questionnaire (DASH) [28] using the validated Brazilian Portuguese version [29]. The final score of the DASH questionnaire will be converted to a percentage via the following formula:

$$ \mathrm{Scoring}\kern0.5em =\kern0.5em \left[\mathrm{Sum}\kern0.5em \mathrm{of}\kern0.5em \mathrm{answers}\kern0.5em n/n-1\right]\kern0.5em \times \kern0.5em 25, $$

where *n* is the number of complete answers. At least 27 of the 30 items must be completed for a score to be calculated. The value is then transformed to a score out of 100. A higher score indicates greater disability. The two optional modules (sport/music or work) will not be measured.

#### Secondary outcomes

1. Modified University of California at Los Angeles (modified - UCLA) [30, 31], validated and translated into Portuguese in Brazil [32]
2. Pain measured on a 0 to 100 Visual Analogue Scale (VAS) (with 0 indicating no pain and 100 indicating maximum pain) [33, 34]. As reported in the literature, a clinically important change will be considered as a 30 % or more change in pain score [35, 36]. The total of analgesic consumed by the patients during the follow-up will be analysed
3. Treatment failure will be considered as outcome in those participants who will need (or are being considered for) a surgical intervention (e.g. symptomatic nonunion or malunion with intractable pain). Although studies evaluating patients with fractures without displacement reported low rates of nonunion (about 0.03 %) [5, 6, 15], studies involving patients with displaced fractures found nonunion rates of up to 15 % [37–39]. Therefore, we expect that between 5 % and 10 % of our participants might exhibit symptomatic nonunion during follow-up period. The patients who experience this complication during follow-up will be treated surgically with open reduction and internal fixation with a pre-contoured locking plate placed on the superior surface of the clavicle, and bone grafting when necessary
4. Adverse events and effects will be evaluated by the following parameters: (a) cosmetic results: perception of deformity or asymmetry (dichotomous data); (b) asymptomatic nonunion (i.e. the fracture has not radiographically healed, although pain is absent); (c) stiffness/restriction of the shoulder movement (compared with contralateral side). We will also address any possible adverse event reported by participants.
  In 1986, the American Food and Drug Administration (FDA) defined nonunion to be ‘established when a minimum of 9 months has elapsed since injury and the fracture shows no visible progressive signs of healing for 3 months’. However, these criteria cannot be applied to every fracture [37]. Even though nonunion of the clavicle has not been definitively defined in the literature so far, many investigators agree that a diagnosis can be made if consolidation does not happen within 6 months after the injury [38–40]. The verification of the nonunion is made when there is clinical or radiographic evidence showing that healing has ceased and that union is highly improbable. We shall apply 6 months as the criterion
5. Numbers returning to previous activities (work, sport, activities of daily living), including time to return

Following enrolment in the study, the participants will be seen by one author (LFPT) at 1, 2 and 4 weeks and 3, 6 and 12 months. All primary and secondary outcomes will be recorded at each time period. Radiographs will be taken until there is evidence of clinical union at 3, 6 and 12 months.

The treatment failures and adverse events will be considered according to their date of occurrence. We will extract adverse event outcome data at the following time periods: short-term follow-up (up to 6 weeks following treatment); intermediate follow-up (more than 6 weeks and up to 6 months after the end of treatment) and long-term follow-up (more than 6 months after the end of treatment).

### Assignment of interventions

All patients will be assessed in the Emergency Room of the Municipal Hospital Dr. Moyses Deutsch, referral hospital for trauma, and will undergo clinical and radiographic examination with anteroposterior radiographs and a cephalic tilt of 20° (Zanca inclination [41]).

#### Randomisation and allocation concealment

In the fracture clinic or emergency room, the orthopaedic resident or general orthopaedic surgeon will identify the eligible participant in accordance with the above inclusion and exclusion criteria and the study protocol will be started. Once informed consent is obtained, participants will be randomised according to computer-generated randomisation (http://www.randomizer.org) by an independent secretary (LA) not involved in the study. Allocation will be sealed in opaque and consecutively numbered envelopes, which will be opened in sequence by LA and will inform the type of intervention (figure-of-eight or arm sling) that the treating orthopaedic surgeon will apply. A person not involved with study will open the envelope before the intervention.

The sealed opaque envelope containing the method of intervention to be applied will be attached to each patient record.

#### Blinding

Due to the type of interventions, neither participants nor treatment providers can be blinded to treatment allocation. The outcome assessment of the primary outcome (DASH), a patient-reported outcome, will not be blind. One author (LFPT) will assess all other clinical outcomes. All primary and secondary outcomes will be assessed at 1, 2 and 4 weeks and 3, 6 and 12 months. Radiographic outcomes will be blinded assessed at 3, 6 and 12 months by two authors (ML and MF). The statisticians conducting the analyses will be blinded to the treatment status until the analyses are completed.

### Data collection and analysis

#### Sample size

Sample size estimations were performed before patient recruitment. The main variable used was the DASH questionnaire. Type I error was pre-established as 5 % (95 % confidence interval) and type II error as 10 % (power of 90 %) with a population standard deviation of 15 %.

In order to calculate our sample size, we used the mathematical method described in the formula below:

$$ \mathrm{n}=\left[{\left(\mathrm{Z}\upalpha /2+\mathrm{Z}\upbeta \right)}^2\times {\upsigma}^2\right]/{\upvarepsilon}^2, $$

where Z*α*/2 is the critical value *α* from the standard normal distribution with upper tail; Z*β* is the critical value *β* from the standard normal distribution; *σ* is population standard deviation; *ε* is the difference in the DASH questionnaire, which was assumed to be clinically relevant (10 points = minimal clinically important difference) [28, 42].

Thus, assuming the values described in the text, we have anticipated that 50 participants would be required in each group. Allowing for approximately 10 % loss to follow-up at 12 months, we aim to recruit a total of 110 patients.

#### Data analysis

Demographic and baseline data will be summarised in the two study groups presenting number in each category and percentages of the number of patients in each group for categorical variables and presenting means and standard deviations or medians and quartiles for continuous variables. If we observe some clinically important imbalance between groups regarding to baseline variables we will adjust for them in the main analysis.

Patients, who for any reason may require additional interventions as part of their treatment, will be followed up, and their results will be included in the group into which they had initially been randomised, according to the intention-to-treat principle.

The primary outcome (DASH score) and the secondary outcomes (modified UCLA and VAS scores) will be analysed graphically. We will apply generalised mixed models in the intervention groups (two levels) and time-point assessments (seven levels) as fixed effects. To evaluate the effect of surgical interventions required due to treatment failure in the DASH score a similar model will be adjusted including the effect of surgery (two levels). In this analysis, patients who are undergoing surgery will have the data analysed up to the last assessment before surgery.

Secondary outcomes: treatment failure, adverse events and return to activities will be analysed individually by the chi-square test. The time to return to previous activities will be analysed by a Kaplan-Meier curve and Cox proportional hazard regression. The total analgesic amount consumed by the patients during the follow-up will be analysed by comparing study groups using the Mann-Whitney test.

To handle the effects of missing data we will describe patterns of missingness in the study groups and use the model-based method of multiple imputation to replace missing data.

The significance level of 5 % will be adopted in the analysis of the primary outcome and 1 % in the secondary outcomes, to avoid chance of statistical significance due to multiple tests.

## Discussion

This protocol describes a pragmatic trial design, which has been chosen because of its direct applicability to clinical practice. Thus, this study includes design characteristics known to minimise bias [43]. Participants will be assigned using a concealed random procedure, assessments and data analysis will be blinded and we will use the intention-to-treat analysis. Consequently, our inclusion criteria will reflect the variety of patient presentations that would be encountered by general orthopaedic surgeons in the clinical setting. In addition, our primary outcome is a validated Patient Reported Outcome Measures (PROMs) for the shoulder.

Currently, acute displaced middle-third clavicle fractures are conventionally treated by conservative interventions with high expectation of fracture union and patient satisfaction. However, a recent multicentre randomised controlled trial demonstrated better functional outcomes with surgical interventions, but the authors did not support routine primary surgical treatment for these fractures [44].

A small randomised controlled trial was recently published [45]; it included 60 participants with an acute, isolated middle-third clavicle fracture. The authors compared the broad arm sling with the figure-of-eight bandage; their outcome measures were pain, Constant and American Shoulder and Elbow Surgeons scores and degree of radiographic union. The study concluded that both techniques provided acceptable functional and radiological outcomes for treating clavicle fractures. However, the broad arm sling was significantly more comfortable in the first 3 days of treatment. One weakness of that study was the small sample size and the additional loss of patients in the final follow-up period (two patients in the broad arm sling group and seven patients in the figure-of-eight bandage group), which may have affected the results, since the sampling loss was greater in the figure-of-eight bandage group. The study had other sources of bias. First, the study design did not permit blinding of participants, orthopaedic surgeons and outcome assessors; although failure of blinding can have a serious effect on study outcomes, the authors were unable to perform it. Secondly, the function outcomes (Constant and American Shoulder and Elbow Surgeons scores) were not validated for use in a number of settings (mainly Constant score) and these scores may not be specific for use as an outcome in clavicle fractures [45].

According to current evidence from a systematic review, there is very limited evidence from two single trials regarding the effectiveness of different methods of conservative interventions for treating clavicle fractures [16]. The reported results in the literature regarding the outcome of conservative treatment of middle-third clavicle fractures are controversial [5, 46]. In addition, as a conclusion of the most updated Cochrane systematic review [16], randomised controlled trials comparing contemporary conservative interventions, such as an arm sling versus the figure-of-eight bandage, for clavicle fractures are warranted. Consequently, we expect that our present study will provide conclusive results that can be readily used in clinical practice and offer evidence-based approaches for the conservative treatment of middle-third clavicle fractures.

## Trial status

This trial started recruiting patients in November 2015.

## Additional files

Additional file 1: Informed consent (Portuguese). (PDF 123 kb) Additional file 2: Informed consent (English). (PDF 128 kb) Additional file 3: Data collection form. (PDF 427 kb)

## Abbreviations

CONSORT: Consolidated Standards of Reporting Trials
DASH: Disability of the Arm, Shoulder and Hand
FDA: American Food and Drug Administration
PROMs: Patient Reported Outcome Measures
UCLA: University of California at Los Angeles
VAS: Visual Analogue Scale
