# Supplementary material for: Figure-of-eight bandage versus arm sling for treating middle-third clavicle fractures in adults: study protocol for a randomised controlled trial
Source: Trials. 2016 May 4;17:229. doi: 10.1186/s13063-016-1355-8 (PMC4855803; doi:10.1186/s13063-016-1355-8)
Supplement: Additional file 1: — Informed consent (Portuguese). (PDF 123 kb) [file 13063_2016_1355_MOESM1_ESM.pdf]

Additional file 1 Informed Consent (Portuguese)

TERMO DE CONSENTIMENTO LIVRE E ESCLARECIDO

***Título do projeto**: Imobilização em oito versus tipoia simples para o tratamento das fraturas do terço médio da clavícula: um ensaio clínico randomizado.*

Prezado Paciente,

Você foi convidado a participar da pesquisa intitulada "Imobilização em oito versus tipoia simples para o tratamento das fraturas do terço médio da clavícula: um ensaio clínico randomizado". Essas informações serão fornecidas por meio de sua participação voluntária neste estudo, com o objetivo de avaliar a efetividade (benefícios e malefícios) das intervenções: imobilização em oito versus uso da tipoia simples para o tratamento de fraturas de clavícula do terço médio da clavícula.

A motivação da realização deste estudo se deve ao fato de encontrarmos na literatura atual dúvidas de qual seja o melhor tratamento conservador para as fraturas da clavícula. Acreditarmos que o tratamento das fraturas da clavícula com o uso de tipoia simples apresente os mesmos resultados funcionais e de qualidade de vida que o tratamento com a imobilização em oito.

Procedimentos a serem utilizados:

O número de participantes estimado é de 110 indivíduos, divididos em dois grupos, 55 para o grupo de imobilização com tipoia simples e 55 para o grupo de imobilização em oito. A seleção será feita de forma randomizada, isto é, não saberemos onde cada indivíduo será incluído.

Os dois grupos de pacientes receberão os mesmos cuidados e os mesmos seguimentos. As avaliações serão realizadas por meio de exames clínicos (realizado por um médico) e exames radiográficos em 1, 2 e 4 semanas e em 3, 6 e 12 meses após a intervenção. O programa de reabilitação após as intervenções de tratamento será idêntico em cada um dos grupos comparados. Depois de 4 semanas de tratamento, um fisioterapeuta instruirá os participantes a realizar alguns exercícios simples de alongamento em casa.

No término do estudo será verificado se houve melhora nas avaliações funcionais, de qualidade de vida e radiográficas por meio de questionários de simples preenchimento e radiografias nas posições ântero-

posterior (AP) e AP com inclinação cranial de 20º das suas clavículas. Não há risco ou desconforto para os pacientes que estarão expostos à radiação usada para avaliação de fraturas da clavícula.

Não haverá benefício para o participante. Trata-se de um estudo experimental, testando a hipótese de que tratamento das fraturas da clavícula com o uso de tipoia simples apresenta os mesmos resultados funcionais e de qualidade de vida quanto o tratamento com a imobilização em oito. Somente no final do estudo poderemos concluir a presença de algum benefício entre estes dois tipos de tratamento (tipoia e imobilização em oito).

Em qualquer etapa do estudo, você terá acesso aos profissionais responsáveis pela pesquisa para esclarecimento de eventuais dúvidas. O coordenador do projeto é o Dr. Mário Lenza e o principal responsável pelo estudo é o Dr. Luiz Fabiano Presente Taniguchi, que podem ser encontrados: Dr. Mario Lenza – endereço Av. Albert Einstein, 627 – bloco A1 – 3º andar – Programa Locomotor, Morumbi, São Paulo – CEP 05652-900: Tel: (11) 2151.1444; e-mail: mario.lenza@einstein.br; e Dr. Luiz Fabiano Presente Taniguchi – endereço Hospital Municipal Dr. Moysés Deutsch - M'Boi Mirim Estrada do M'Boi Mirim, 5203 – Ortopedia, Jd. Ângela, São Paulo – CEP 04948-970: Tel: (11) 5832-2500; e-mail: Luiz.Taniguchi@hmbm.org.br.

Se você tiver qualquer dúvida ética em relação à pesquisa, entre em contato com o Comitê de ética em Pesquisa da Secretaria Municipal de Saúde de São Paulo, Rua General Jardim nº36, 1º Andar, Fone 3397-2464, e-mail: smscep@gmail.com, ou com o Comitê de Ética em Pesquisa do Hospital Israelita Albert Einstein Av. Albert Einstein 627/701, fone 2151-0291, e-mail: cep@einstein.br.

É garantida a liberdade da retirada de consentimento a qualquer momento e deixar de participar do estudo, sem qualquer prejuízo à continuidade de seu tratamento na Instituição.

As informações obtidas serão analisadas em conjunto com outros pacientes voluntários, não sendo divulgada a identificação de nenhum paciente. Você tem o direito de ser mantido atualizado sobre os resultados parciais das pesquisas e de quaisquer resultados que sejam do conhecimento dos pesquisadores. Não há despesas pessoais para o participante em qualquer fase do estudo, incluindo exames e consultas. Também não há compensação financeira relacionada à sua participação. Se existir qualquer despesa adicional, ela será absorvida pelo orçamento da pesquisa.

Na improvável ocorrência de algum dano pessoal, diretamente causado pelos procedimentos ou tratamentos propostos neste estudo (nexo causal comprovado), o participante terá o direito a tratamento médico na instituição, bem como às indenizações legalmente estabelecidas.

O pesquisador tem o compromisso de utilizar os dados e materiais coletado durante o estudo, somente para esta pesquisa.

Acredito ter sido suficientemente informado a respeito das informações que li ou que foram lidas para mim e assinando este documento concordo em participar deste estudo.

Assinatura do paciente/representante legal	Nome completo do paciente/representante legal<br>Data __/__/__

Assinatura da testemunha*	Nome completo da testemunha<br>Data __/__/__

*para casos de pacientes menores de 18 anos, analfabetos, semi-analfabetos ou portadores de deficiência auditiva ou visual.

(<u>Somente para o responsável do projeto</u>)

Declaro que obtive de forma apropriada e voluntária o Consentimento Livre e Esclarecido deste paciente ou representante legal para a participação neste estudo.

Assinatura do responsável pelo estudo	Nome complete do responsável pelo estudo<br>Data __/__/__
