# Supplementary material for: Figure-of-eight bandage versus arm sling for treating middle-third clavicle fractures in adults: study protocol for a randomised controlled trial
Source: Trials. 2016 May 4;17:229. doi: 10.1186/s13063-016-1355-8 (PMC4855803; doi:10.1186/s13063-016-1355-8)
Supplement: Additional file 2: — Informed consent (English). (PDF 128 kb) [file 13063_2016_1355_MOESM2_ESM.pdf]

Additional file 2 Informed Consent (English)

SUBJECT INFORMATION AND CONSENT FORM

Title of study: Figure-of-eight bandage versus arm sling for treating middle third clavicle fractures in adults: study protocol for a randomised controlled trial

Dear Patient,

You are being asked to participate in a Clinical Trial entitled "Figure-of-eight bandage versus arm sling for treating middle third clavicle fractures in adults: study protocol for a randomised controlled trial". Please take your time to review this consent form and discuss any questions you may have with the study staff. The purpose of this study is to assess the effects (benefits and harms) of interventions: figure-of-eight bandage versus arm sling for treating middle third clavicle fractures.

This research is being done because we found no consensus in the literature about the best conservative treatment for clavicle fractures. We believe that treatment of clavicle fractures with simple sling shows similar functional and quality of life outcomes that treatment with figure-of-eight bandage.

Study procedures:

About 110 people will take part in this study divided into two groups, 55 for using simple sling and 55 for using figure-of-eight bandage. Selection will be made randomly, that is, we will not know where each individual will be included. Randomisation means that you are put into a group by chance. A computer program will place you in one of the study groups. Neither you nor your doctor can select the group you will be in. You will have an identical chance of being placed in either group.

The two groups of patients will be given the same care and the same follow-up. Evaluations shall be performed by clinical examination and X-rays in 1, 2 and 4 weeks and at 3, 6 and 12 months after the intervention. The rehabilitation program after treatment interventions will be identical in each of the compared groups. After 4 weeks of treatment, a physical therapist will instruct participants to perform some simple stretching exercises at home.

At the end of the study will be checked if there was an improvement in functional assessments, quality of life and radiographic image through simple questionnaires filling and radiographs in anteroposterior position (AP) and AP with cephalic tilt of approximately 15 degrees. There is no risk or discomfort to patients who are exposed to the radiation used by these X-rays.

By participating in this study, you will be providing information to the study doctors that will show the effects of simple sling and figure-of-eight bandage for the treatment of clavicle fractures. There may or may not be direct medical benefit to you from participating in this study. We hope the information learned from this study will benefit other participants with clavicle fractures in the future.

All clinic and professional fees, diagnostic and radiographic tests which will be performed as part of this study are provided at no cost to you. There will be no cost for the study treatment that you will receive. You will receive no payment or reimbursement for any expenses related to taking part in this study.

At any stage of the study, you will have access to professionals responsible for this research to clarify any doubts. The project coordinator is Dr. Mario Lenza and the main author of the study is Dr. Luiz Fabiano Gift Taniguchi, who can be found: Dr. Mario Lenza – Address: Av Albert Einstein, 627 bloco A1 – 3rd floor – Programa Locomotor, Morumbi, São Paulo – CEP 05652-900, Phone: (11) 2151.1444, e-mail: mario.lenza@einstein.br; and Dr. Luiz Fabiano Presente Taniguchi – Address: Hospital Municipal Dr. Moysés Deutsch - M'Boi Mirim Estrada do M'Boi Mirim, 5203 – Ortopedia, Jd. Angela, São Paulo – CEP 04948-970, Phone: (11) 5832-2500, e-mail: Luiz.Taniguchi@hmbm.org.br.

This consent form may contain words that you do not understand. Please ask the study doctor or study staff to explain any words or information that you do not clearly understand. If you have any ethical questions regarding the research, please contact the Ethics in Research Committee of the Municipal Health of São Paulo - Comitê de ética em Pesquisa da Secretaria Municipal de Saúde de São Paulo, Rua General Jardim nº36, 1st floor, phone 3397-2464, email: smscep@gmail.com, or the Research Ethics Committee of the Hospital Israelita Albert Einstein - Comitê de Ética em Pesquisa do Hospital Israelita Albert Einstein - Av. Albert Einstein 627/701, phone 2151-0291, e-mail: cep@einstein.br.

Your decision to take part in this study is voluntary. You may refuse to participate or you may withdraw from the study at any time. Your decision not to participate or to withdraw from the study will not affect your other medical care at this site.

Information gathered in this research study may be published or presented in public forums; however your name and other identifying information will not be used or revealed. Medical records that contain your identity will be treated as confidential.

If you should become physically injured as a result of any research activity, the study doctor will provide any necessary treatment, at no charge, to help you promptly recover from the injury, as well as legally established damages.

I have read this consent form. I understand that my participation in this clinical trial is voluntary and that I may choose to withdraw at any time. I freely agree to participate in this research study.

______________________________________	____
Participant or parent/legal guardian's signature	Participant or parent/legal guardian's printed name<br>Date __/__/__

______________________________________	____
Witness signature*	Witness printed name<br>Date __/__/__

*For cases of patients semi-illiterate or people with hearing or visual impairment.

(<u>Only the head of the study</u>)

I declare that I got properly and voluntarily the Informed Consent of the patient or legal representative to participate in this study.

______________________________________	____
Coordinator of study signature	Coordinator of study printed name<br>Date __/__/__
