# Supplementary material for: Figure-of-eight bandage versus arm sling for treating middle-third clavicle fractures in adults: study protocol for a randomised controlled trial
Source: Trials. 2016 May 4;17:229. doi: 10.1186/s13063-016-1355-8 (PMC4855803; doi:10.1186/s13063-016-1355-8)
Supplement: Additional file 3: — Data collection form. (PDF 427 kb) [file 13063_2016_1355_MOESM3_ESM.pdf]

Additional file 3 Data collection form

Ficha de coleta de dados dos pacientes (*Pre-piloted data extraction form*)

Título do Projeto: *Imobilização em oito versus tipoia simples para o tratamento das fraturas do terço médio da clavícula: um ensaio clínico randomizado.*

***Title**: Figure of eight bandage versus arm sling for treating middle third clavicle fractures in adults: a randomised controlled trial.*

Coordenador do Projeto *(Coordinator):* Mario Lenza

Médico Responsável (*Physician guarantor*): Luiz Fabiano Presente Taniguchi

Número do paciente no estudo (*Number of patient in the study*): ___________________.

Data do início (*Date*): __ / __ / ____.

Nome (*Name*): ____.

Registro hospitalar (*Medical records*): ____.

Telefones (*Phones*): ___________________; ___________________; __________________.

Endereço (Address): ____.

____.

____.

Email: ____.

Data de nascimento (*Birth date*): __ / __ / ____

Idade (*age*): ___ anos (*years*)

Gênero (*Gender*): ( ) masculino (*male*) ( ) feminino (*female*)

Altura (*Height*): ____ cm Peso (*Weight*): ____ kg

Braço dominante (*Dominant arm*): ( ) direito (*right*) ( ) esquerdo (*left*)

Lado da lesão (*Side of injury*): ( ) direito (*right*) ( ) esquerdo (*left*)

Tabagismo (*Smoker*): ( ) sim (*yes*) ( ) não (*no*)

Comorbidades (*comorbidities*):

( ) Diabetes (*Diabetes*)

( ) Hipertensão (*Hypertension*)

( ) Outras (*Other*): ____.

Mecanismo da lesão (*Mechanism of injury*):

( ) Queda simples (*Simple fall*) 22 22

( ) Acidente automobilístico (*Road traffic accident*)

( ) Esporte (*Sport*)

( ) Bicicleta/moto (*Bicycle/motorcycle*)

( ) Outros (*Other*): ____.

Cominuição (*Comminution*): ( ) sim (*yes*) ( ) não (*no*)

Deslocamento máximo (*Maximum displacement*): ____ mm

Nível de participação em esportes (*Level of participation in sports*):

(   ) Competitivo (*Competitive*)

(   ) Social (*Social*)

(   ) Nenhum (*None*)

Tipo de trabalho (*Employment status*):

(   ) Manual/Braçal (*Manual labor*)

(   ) Sedentário (*Sedentary*)

(   ) Desempregado (*Unemployed*)

Classificação da lesão (*Injury classification*):

- Por favor, assinale a classificação mais apropriada (*Please check the most appropriated fracture*):

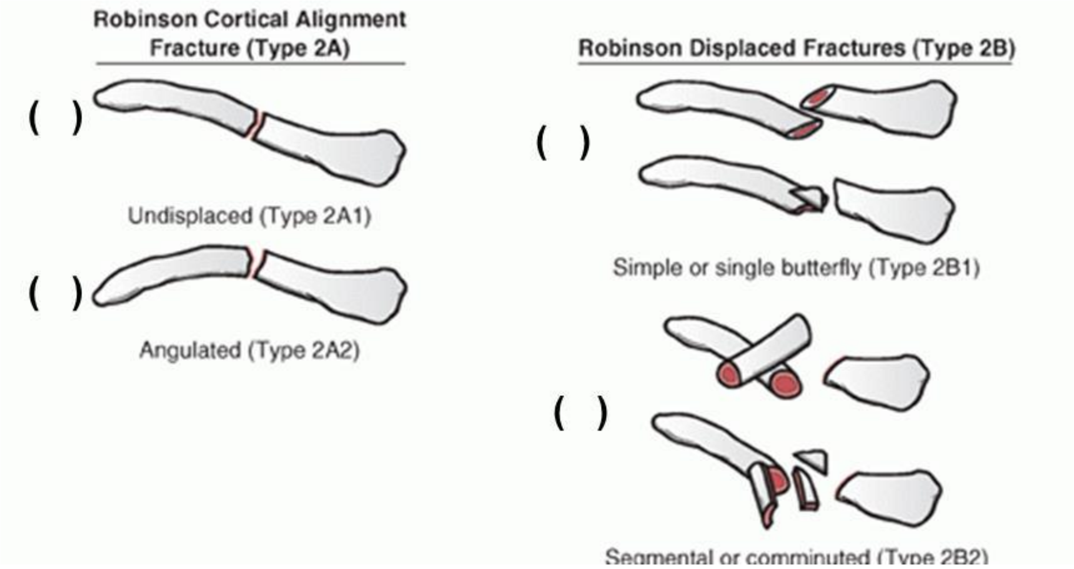

Desfecho – (outcome):

DASH[27,28]**:**

Instruções

Esse questionário é sobre seus sintomas, assim como suas habilidades para fazer certas atividades.
Por favor, responda a todas as questões baseando-se na sua condição na semana passada.
Se você não teve a oportunidade de fazer uma das atividades na semana passada, por favor, tente estimar qual resposta seria a mais correta.
Não importa qual mão ou braço você usa para fazer a atividade; por favor, responda baseando-se na sua habilidade independentemente da forma como você faz a tarefa.

Meça a sua habilidade em fazer as seguintes atividades na semana passada circulando a resposta apropriada abaixo:

	Não houve dificuldade	Houve pouca dificuldade	Houve dificuldade média	Houve muita dificuldade	Não conseguiu fazer
1. Abrir um vidro novo ou com a tampa muito apertada	1	2	3	4	5
2. Escrever	1	2	3	4	5
3. Virar uma chave	1	2	3	4	5
4. Preparar uma refeição	1	2	3	4	5
5. Abrir uma porta pesada	1	2	3	4	5
6. Colocar algo em uma prateleira acima de sua cabeça	1	2	3	4	5
7. Fazer tarefas domésticas pesadas (por exemplo: lavar paredes, lavar o chão)	1	2	3	4	5
8. Fazer trabalho de jardinagem	1	2	3	4	5
9. Arrumar a cama	1	2	3	4	5
10. Carregar uma sacola ou uma maleta	1	2	3	4	5
11. Carregar um objeto pesado (mais de 5 kg)	1	2	3	4	5
12. Trocar uma lâmpada acima da cabeça	1	2	3	4	5
13. Lavar ou secar o cabelo	1	2	3	4	5
14. Lavar suas costas	1	2	3	4	5
15. Vestir uma blusa fechada	1	2	3	4	5
16. Usar uma faca para cortar alimentos	1	2	3	4	5
17. Atividades recreativas que exigem pouco esforço (por exemplo: jogar cartas, tricotar)	1	2	3	4	5
18. Atividades recreativas que exigem força ou impacto nos braços, ombros ou mãos (por exemplo: jogar vôlei, martelar)	1	2	3	4	5
19. Atividades recreativas nas quais você move seu braço livremente (como pescar, jogar peteca)	1	2	3	4	5
20. Transportar-se de um lugar a outro (ir de um lugar a outro)	1	2	3	4	5
21. Atividades sexuais	1	2	3	4	5

	Não afetou	Afetou pouco	Afetou medianamente	Afetou muito	Afetou extremamente
22. Na semana passada, em que ponto o seu problema com braço, ombro ou mão afetou suas atividades normais com família, amigos, vizinhos ou colegas?	1	2	3	4	5

	Não limitou	Limitou pouco	Limitou medianamente	Limitou muito	Não conseguiu fazer
23. Durante a semana passada, o seu trabalho ou atividades diárias normais foram limitadas devido ao seu problema com braço, ombro ou mão?	1	2	3	4	5

Meça a gravidade dos seguintes sintomas na semana passada:	Nenhuma	Pouca	Mediana	Muita	Extrema
24. Dor no braço, ombro ou mão	1	2	3	4	5
25. Dor no braço, ombro ou mão quando você fazia atividades específicas	1	2	3	4	5
26. Desconforto na pele (alfinetadas) no braço, ombro ou mão	1	2	3	4	5
27. Fraqueza no braço, ombro ou mão	1	2	3	4	5
28. Dificuldade em mover braço, ombro ou mão	1	2	3	4	5

	Não houve dificuldade	Pouca dificuldade	Média dificuldade	Muita dificuldade	Tão difícil que você não pôde dormir
29. Durante a semana passada, qual a dificuldade que você teve para dormir por causa da dor no seu braço, ombro ou mão?	1	2	3	4	5

	Discordo totalmente	Discordo	Não concordo nem discordo	Concordo	Concordo totalmente
30. Eu me sinto menos capaz, menos confiante e menos útil por causa do meu problema com braço, ombro ou mão	1	2	3	4	5

Total (Total): ________

Desfecho (outcome):

Modified University of California at Los Angeles (modified - UCLA)[29-31]

DOR

1) Presente todo o tempo e insuportável; faz uso de medicação regularmente	1
2) Presente todo o tempo, mas suportável; faz uso de medicação de vez em quando	2
3) Nenhuma ou pouca dor quando o braço está parado, ocorre durante trabalhos leves; faz uso de medicação regularmente	4
4) Ocorre apenas durante trabalhos pesados ou durante trabalhos específicos; faz uso de medicação de vez em quando	6
5) Dor leve e ocorre de vez em quando	8
6) Nenhuma	10

FUNÇÃO

1) Incapaz de usar o braço	1
2) Capaz apenas de realizar atividades leves	2
3) Capaz de realizar trabalhos domésticos leves ou a maioria dos trabalhos do dia-a-dia	4
4) Capaz de realizar a maioria dos trabalhos domésticos, inclusive fazer compras, dirigir, pentear-se, vestir-se, despir-se e fechar o sutiã	6
5) Apresenta pouca dificuldade, capaz de realizar movimentos acima da altura do ombro	8
6) Atividades normais	10

Instruções para goniometria

O paciente deve se posicionar sentado com o membro ao lado do corpo e posição neutra. O examinador deve instrui-lo a elevar o braço o máximo possível sem compensações.

O goniômetro será posicionado com braço proximal na linha média axilar do tórax e braço distal na linha médio lateral do úmero, sendo o eixo colocado próximo ao acrômio.

FLEXÃO ANTERIOR ATIVA

1) 150 graus ou mais	5
2) 120 a 150 graus	4
3) 90 a 120 graus	3
4) 45 a 90 graus	2
5) 30 a 45 graus	1
6) Menos que 30 graus	0

Instruções para o teste de força manual

O paciente deve se posicionar sentado com o membro ao lado do corpo e antebraço pronado, a seguir deve elevar o membro a 90 graus. O examinador devera instrui-lo a manter esta posição contra resistência que será aplicada na porção distal do úmero (acima do cotovelo).

FORÇA DE FLEXÃO ANTERIOR ATIVA
(TESTE DE FORÇA MANUAL)

1) Grau 5 (normal)	5
2) Grau 4 (boa)	4
3) Grau 3 (regular)	3
4) Grau 2 (fraco)	2
5) Grau 1 (contração muscular)	1
6) Grau 0 (ausência de contração)	0

SATISFAÇÃO DO PACIENTE

1) Satisfeito e melhor	5
2) Insatisfeito e pior	0

Total (Total): ____

Desfecho (outcome):

EVA (VAS)[32-35]

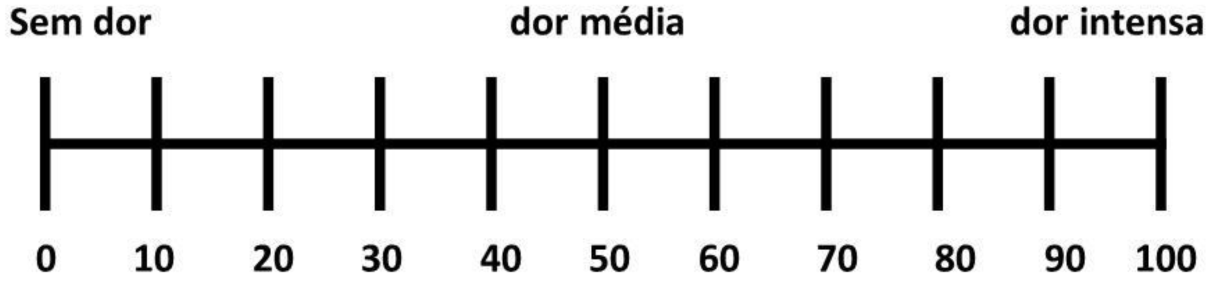

Desfecho (outcome):
Falha do tratamento (Treatment failure): ( ) sim (*yes*) ( ) não (*no*)

Eventos adversos mensurados por:

Resultado cosmético (Cosmetic result): ( ) sim (*yes*) ( ) não (*no*)

Pseudartrose assintomática (Asymptomatic non-union): ( ) sim (*yes*) ( ) não (*no*)

Arco de movimento (Range of motion):
Elevação (Elevation):
lado com lesão (injury side): ____ lado sem lesão (non-injury side): ____

Rotação lateral (Lateral rotation):
lado com lesão (injury side): ____ lado sem lesão (non-injury side): ____

Rotação medial (Medial rotation):
lado com lesão (injury side): ____ lado sem lesão (non-injury side): ____

Retorno às atividades (Returning to activities): ( ) sim (*yes*) ( ) não (*no*)
Se sim, quanto tempo após a fratura (if yes, for how long after fracture): ________.
